# Modeling the Parasitic Filariasis Spread by Mosquito in Periodic Environment

**DOI:** 10.1155/2017/4567452

**Published:** 2017-02-08

**Authors:** Yan Cheng, Xiaoyun Wang, Qiuhui Pan, Mingfeng He

**Affiliations:** ^1^School of Mathematics, Taiyuan University of Technology, Taiyuan 030024, China; ^2^School of Innovation Experiment, Dalian University of Technology, Dalian 116024, China

## Abstract

In this paper a mosquito-borne parasitic infection model in periodic environment is considered. Threshold parameter *R*_0_ is given by linear next infection operator, which determined the dynamic behaviors of system. We obtain that when *R*_0_ < 1, the disease-free periodic solution is globally asymptotically stable and when *R*_0_ > 1 by Poincaré map we obtain that disease is uniformly persistent. Numerical simulations support the results and sensitivity analysis shows effects of parameters on *R*_0_, which provided references to seek optimal measures to control the transmission of lymphatic filariasis.

## 1. Introduction

Lymphatic filariasis is a parasitic disease caused by filarial nematode worms and is a mosquito-borne disease that is a leading cause of morbidity worldwide. Lymphatic filariasis affects 120 million humans in tropical and subtropical areas of Asia, Africa, the Western Pacific, and some parts of the Americas [1]. It is estimated that 40 million people are chronically disabled by lymphatic filariasis, making lymphatic filariasis the leading cause of physical disability in the world [2]. There are some clinical manifestations for infective individuals, such as acute fevers, chronic lymphedema, elephantiasis, and hydrocele [3].

*W. bancrofti* parasites, which account for 90% of the global disease burden, dwell in the lymphatic system, where the adult female worms release microfilariae (mf) into the blood. Mf are ingested by biting mosquitoes as a blood meal of a mosquito, through several developmental stages, that is, first into immature larvae and then L3 larvae. Infective stage larvae L3 actively escape from the mosquito mouthparts entering another human host at the next blood meal through skin [4]. These L3 larvae subsequently develop into worms in humans and the process continues. So in order to remove lymphatic filariasis from the society, not only are the infected persons to be recovered but also the infected vectors are to be killed or removed.

Mathematical models are powerful tools in disease control and may provide a powerful strategic tool for designing and planning control programs against infectious diseases [5]. Since 1960s, simple mathematical models of infection have been in existence for filariasis and provided useful insights into the dynamics of infection and disease in human populations [6–8]. Michael et al. describe the first application of the moment closure equation approach to model the sources and the impact of this heterogeneity for microfilarial population dynamics [9]. Simulation model for lymphatic filariasis transmission and control [10, 11] suggests that the impact of mass treatment depends strongly on the mosquito biting rate and on the assumed coverage, compliance, and efficacy; sensitivity analysis showed that some biological parameters strongly influence the predicted equilibrium pretreatment mf prevalence. References [12–14] take into account the complex interrelationships between the parasite and its human and vector hosts and provide the management decision support framework required for defining optimal intervention strategies and for monitoring and evaluating community-based interventions for controlling or eliminating parasitic diseases. Gambhir and Michael have shown a joint stability analysis of the deterministic filariasis transmission model [15]. All such models have proved to be of great value in guiding and assessing control efforts [16, 17].

Environmental and climatic factors play an important role for the transmission of vector-borne diseases and are researched in many articles [18, 19]. For lymphatic filariasis, proper temperature and humidity are more beneficial for mosquito population to give birth and propagate. For example, in temperate climates and in tropical highlands, temperature restricts vector multiplication and the development of the parasite in the mosquito, while in arid climates precipitation restricts mosquito breeding. Therefore, the transmission of lymphatic filariasis exhibits seasonal behaviors especially in the northern areas [20, 21]. Nonautonomous phenomenon in infectious disease often occurs, and basic reproductive number of periodic systems is described as the spectral radius of the next infection operator [22].

But the dynamics system considers the periodic environment between human and mosquito is little. How to make a comprehensive understanding of the role of periodic environment in the transmission of lymphatic filariasis and how to control the transmission of lymphatic filariasis efficiently are problems that provide motivation for our study. For the limitation of ecology environmental resources such as food and habitat, it is reasonable to adopt logistic growth for mosquito population. Nonautonomous logistic equations have been studied [23–28]. Based on above works and [29–34], we investigate a simple lymphatic filariasis model in periodic environment:

(1) Sh′t=Λt−β1tShtImt1+α1tSht−μ1tSht+υtIht,Ih′t=β1tShtImt1+α1tSht−μ1tIht−υtIht,Sm′t=rtSmt1−SmtKt−β2tSmtIht,Im′t=β2tSmtIht−μ2tImt.

In view of the biological background, system (1) has initial values

(2) Sh00>0,Ih00>0,Sm00>0,Im00>0,

where *S*_*h*_(*t*) and *I*_*h*_(*t*) separately denote the densities of the susceptible and the infective individuals for human population at time *t*; *S*_*m*_(*t*) and *I*_*m*_(*t*) represent the densities of the susceptible and the infected individuals for mosquito population at time *t*, respectively. It is easy to see that *N*_*h*_(*t*) = *S*_*h*_(*t*) + *I*_*h*_(*t*) and *N*_*m*_(*t*) = *S*_*m*_(*t*) + *I*_*m*_(*t*) are size of human population and mosquito population, respectively. Λ(*t*) is the recruitment rates of human host at time *t*; *μ*_1_(*t*) and *μ*_2_(*t*) are the death rate of human host and infected mosquito, including the natural death rate and disease-induced death rate; *β*_1_(*t*) and *β*_2_(*t*) denote the contact rate of infected mosquito to humans or infected humans to mosquito; *α*_1_(*t*) is the force of infection saturation at time *t*; *υ*(*t*) is the recovery rate of infectious human host at time *t*; *r*(*t*) and *K*(*t*) are the intrinsic growth rate and the carrying capacity of environment for mosquito population at time *t*, respectively.

In view of the biological background of system (1), we introduce the following assumptions:

(*H*_*1*_) All coefficients are continuous, positive *ω*-periodic functions;
(*H*_*2*_) ∫_0_^*ω*^*r*(*t*)*dt* > 0.

The organization of this paper is as follows. In Section 2, some preliminaries are given and compute the basic production number. In Section 3, we will study the globally asymptotical stability of the disease-free periodic solution and the uniform persistence of the model. In Section 4, simulations and sensitive analysis are given to illustrate theoretical results and exhibit different dynamic behaviors.

## 2. Basic Reproduction Number

Denote

(3) fL=supt∈0,ωft,fM=inft∈0,ωft,

where *f*(*t*) is a continuous *ω*-periodic function.

Let (*R*^*k*^, *R*_+_^*k*^) be the standard ordered *k*-dimensional Euclidean space with a norm ‖·‖. For *u*, *v* ∈ *R*^*k*^, we denote *u* ≥ *v* if *u* − *v* ∈ *R*_+_^*k*^,  *u* > *v* if *u* − *v* ∈ *R*_+_^*k*^∖{0}, and *u* ≫ *v* if *u* − *v* ∈ Int(*R*_+_^*k*^), respectively.

Let *A*(*t*) be a continuous, cooperative, irreducible, and *ω*-periodic *k* × *k* matrix function; we consider the following linear system:

(4) $$\begin{array}{c} \frac{dx\left(t\right)}{dt}=A\left(\;t\;\right)x\left(\;t\;\right). \end{array}$$

Denote Φ_*A*_(*t*) be the fundamental solution matrix of (4) and let *ρ*(Φ_*A*_(*ω*)) be the spectral radius of Φ_*A*_(*ω*). Then by the Perron-Frobenius theorem, *ρ*(Φ_*A*_(*ω*)) is the principle eigenvalue of Φ_*A*_(*ω*) in the sense that it is simple and admits an eigenvector *V*^*∗*^ ≫ 0.

**Lemma 1 (see [35]).**

Let *p* = (1/*ω*)ln⁡*ρ*(Φ_*A*_(*ω*)), where *A*(*t*) is a continuous, cooperative, irreducible, and *ω*-periodic *k* × *k* matrix function. Then system (4) gives a solution *x*(*t*) = *e*^*pt*^*v*(*t*), where *v*(*t*) is a positive *ω*-periodic function.

When system (1) gives disease-free solution, obviously *I*_*h*_(*t*) ≡ 0 and *I*_*m*_(*t*) ≡ 0. So we get the following subsystem:

(5) Sh′t=Λt−μ1tSht,

(6) Sm′t=rtSmt1−SmtKt.

From Lemma 2.1 of [33] and Lemma 2 of [23] we obtain the following lemma.

**Lemma 2 .**

(i) System (5) has a unique positive *ω*-periodic solution *S*_*h*_^*∗*^(*t*) which is globally asymptotically stable. (ii) System (6) has a globally uniformly attractive *ω*-periodic solution *S*_*m*_^*∗*^(*t*).

So, according to Lemma 2, system (1) has a unique disease-free periodic solution (*S*_*h*_^*∗*^(*t*), 0,0, *S*_*m*_^*∗*^(*t*)).

In the following, we use the generation operator approach to define the basic reproduction number of (1). We check the assumptions (A1)–(A7) in [22] and denote *x* = (*I*_*h*_(*t*), *I*_*m*_(*t*), *S*_*h*_(*t*), *S*_*m*_(*t*))^*T*^ and

(7) Ft,x=β1tShtImt1+α1tShtβ2tSmtIht00,V−t,x=μ1tIht+υtIhtμ2tImtβ1tShtImt1+α1tSht+μ1tShtrtSm2tKt+β2tSmtIht,V+t,x=00Λt+υtIhtrtSmt.

So system (1) can be written as the following form:

(8) $$\begin{array}{c} x^{\prime }\left(\;t\;\right)=F\left(\;t,x\left(\;t\;\right)\;\right)-V\left(\;t,x\left(\;t\;\right)\;\right)\equiv f\left(\;t,x\left(\;t\;\right)\;\right), \end{array}$$

where *𝒱*(*t*, *x*) = *𝒱*^−^(*t*, *x*) − *𝒱*^+^(*t*, *x*). From the expressions of *ℱ*(*t*, *x*) and *𝒱*(*t*, *x*), it is easy to see that conditions (A1)–(A5) are satisfied. We will check (A6) and (A7).

Obviously, *x*^*∗*^(*t*) = (0,0, *S*_*h*_^*∗*^(*t*), *S*_*m*_^*∗*^(*t*)) is disease-free periodic solution of system (8). We define

(9) $$\begin{array}{c} M\left(\;t\;\right)={\left(\;\frac{\partial f_{i}\left(t,x^{*}\left(t\right)\right)}{\partial x_{j}}\;\right)}_{3\leq i,j\leq 4}, \end{array}$$

where *f*_*i*_(*t*, *x*^*∗*^(*t*)) and *x*_*i*_ are the *i*th component of *f*(*t*, *x*(*t*)) and *x*, respectively. So we can get

(10) $$\begin{array}{c} M\left(\;t\;\right)={\left(\;\begin{array}{cc} -\mu_{1}\left(t\right) & 0\\ 0 & r\left(t\right)-\frac{2r\left(t\right)}{K\left(t\right)}S_{m}^{*}\left(t\right) \end{array}\;\right)}_{3\leq i,j\leq 4}. \end{array}$$

For *S*_*m*_^*∗*^(*t*) is the globally uniformly attractively *ω*-periodic solution of (6),

(11) $$\begin{array}{c} \overset{\omega }{\underset{0}{\int }}r\left(\;t\;\right)\left[\;1-\frac{S_{m}^{*}\left(t\right)}{K\left(t\right)}\;\right]dt=0. \end{array}$$

Hence,

(12) exp⁡∫0ωrt−2rtKtSm∗tdt=exp⁡−∫0ωrtKtSm∗tdt<1.

It is easy to see that *ρ*(Φ_*M*_(*ω*)) < 1, and condition (A6) holds.

Further, we define

(13) Ft=∂Fit,x∗t∂xj1≤i,j≤2,Vt=∂Vit,x∗t∂xj1≤i,j≤2.

*ℱ*_*i*_(*t*, *x*^*∗*^(*t*)) and *𝒱*_*i*_(*t*, *x*^*∗*^(*t*)) are the *i*th component of *ℱ*(*t*, *x*^*∗*^(*t*)) and *𝒱*(*t*, *x*^*∗*^(*t*)). So we obtain that

(14) Ft=0β1tSh∗t1+α1tSh∗tβ2tSm∗t0,Vt=μ1t+υt00μ2t.

Obviously *ρ*(Φ_−*V*_(*ω*)) < 1; thus condition (A7) holds.

Let *Y*(*t*, *s*) be 2 × 2 matrix solution of the following initial value problem:

(15) $$\begin{array}{c} \frac{dY\left(t,s\right)}{dt}=-V\left(\;t\;\right)Y\left(\;t,s\;\right)\;\forall t\geq s;\;\;Y\left(s,s\right)=I. \end{array}$$

*I* is identity matrix. Let *C*_*ω*_ be the ordered Banach space of all *ω*-periodic functions from *R* → *R*^2^, which is equipped with maximum norm ‖·‖_*∞*_ and the positive cone *C*_*ω*_^+^ = {*ϕ* ∈ *C*_*ω*_ : *ϕ*(*t*) ≥ 0, ∀*t* ∈ *R*}. By the approach in [22], we consider the following linear operator *L* : *C*_*ω*_ → *C*_*ω*_. Suppose that *ϕ*(*s*) ∈ *C*_*ω*_ is the initial distribution of infectious individuals in this periodic environment. *F*(*s*)*ϕ*(*s*) is the distribution of new infections produced by the infected individuals who were introduced at time *s*, and *Y*(*t*, *s*)*F*(*s*)*ϕ*(*s*) represents the distributions of those infected individuals who were newly infected at time s and remain in the infected compartment at time *t*. Then

(16) ψt∫−∞0Yt,sFsϕsds=∫0+∞Yt,t−aFt−aϕt−ada

denotes the distribution of accumulative new infections at time *t* produced by all those infected individuals *ϕ*(*s*) introduced at previous time to *t*.

(17) Lϕt=∫0+∞Yt,t−aFt−aϕt−ada,∀t∈R,  ϕ∈Cω.

As in [22], *L* is the next infection operator, and the basic reproduction number of system (1) is given by

(18) $$\begin{array}{c} R_{0}=\rho \left(\;L\;\right), \end{array}$$

where *ρ*(*L*) is the radius of *L*. Next we show that *R*_0_ serves as a threshold parameter for the local stability of the disease-free periodic solution.

**Theorem 3 (see Wang and Zhao [22], Theorem 2.2).**

Assume that (A1)–(A7) hold; then the following statements are valid:

- *R*_0_ = 1 if and only if *ρ*(Φ_*F*−*V*_(*ω*)) = 1;
- *R*_0_ > 1 if and only if *ρ*(Φ_*F*−*V*_(*ω*)) > 1;
- *R*_0_ < 1 if and only if *ρ*(Φ_*F*−*V*_(*ω*)) < 1.

So the disease-free periodic solution (*S*_*h*_^*∗*^(*t*), 0,0, *S*_*m*_^*∗*^(*t*)) is asymptotically stable if *R*_0_ < 1 and unstable if *R*_0_ > 1.

## 3. Global Stability of Disease-Free Periodic Solution

Denote

(19) Ω=Sh,Ih,Sm,Im:Sh>0,  Ih≥0,  Sm≥0,  Im≥0,  0<Sh+Ih≤ΛLμ1M<+∞,  0≤Sm+Im≤Mm∗Δ<+∞.

*Ω* is a positively invariant set with respect to system (1) and a global attractor of all positive solutions of system (1).

(20) $$\begin{array}{c} N_{h}^{\prime }\left(\;t\;\right)=\Lambda \left(\;t\;\right)-\mu_{1}\left(\;t\;\right)N_{h}\left(\;t\;\right)\leq \Lambda^{L}-\mu_{1}^{M}N_{h}\left(\;t\;\right), \end{array}$$

where Λ^*L*^ = sup_*t*>0_Λ(*t*) and *μ*_1_^*M*^ = inf_*t*>0_*μ*_1_(*t*). So it is easy to obtain *N*_*h*_(*t*) ≤ Λ^*L*^/*μ*_1_^*M*^.

(21) Nm′trtSmt1−SmtKt−μ2tImt≤rt+μ2tSmt−μ2tNmt≤Mm−μ2tNmt,

where *M*_*m*_ = sup_*t*∈[0,*ω*)_(*r*(*t*) + *μ*_2_(*t*))*S*_*m*_(*t*).

From the third equation of (1), for all *t* ≥ 0 we have

(22) $$\begin{array}{c} S_{m}^{\prime }\left(\;t\;\right)\leq r\left(\;t\;\right)S_{m}\left(\;t\;\right)\left(\;1-\frac{S_{m}\left(t\right)}{K\left(t\right)}\;\right); \end{array}$$

by the comparison principle and Lemma 2, we obtain

(23) $$\begin{array}{c} \underset{t\to \infty }{lim}\sup S_{m}\left(\;t\;\right)\leq \underset{t\to \infty }{lim}\sup S_{m}^{*}\left(\;t\;\right)\leq M_{m}^{*}, \end{array}$$

where *S*_*m*_^*∗*^(*t*) is the globally uniformly attractively positive *ω*-periodic solution and *M*_*m*_^*∗*^ = max_*t*∈[0,*ω*]_*S*_*m*_^*∗*^(*t*). So, for any small *ϵ* existing a *t*_0_, for all *t* ≥ *t*_0_ we have

(24) $$\begin{array}{c} S_{m}\left(\;t\;\right)\leq S_{m}^{*}\left(\;t\;\right)+\varepsilon \leq M_{m}^{*}+\epsilon . \end{array}$$

So we obtain

(25) Nm′t≤supt≥0rt+μ2tMm∗+ϵ−μ2tNmt,

and lim_*t*→*∞*_sup⁡*N*_*m*_(*t*) ≤ (*M*_*m*_^*∗*^ + *ϵ*)Δ, where Δ = sup_*t*>0_ (*r*(*t*) + *μ*_2_(*t*))/inf_*t*>0_*μ*_2_(*t*). For *ϵ* small enough, *N*_*m*_(*t*) ≤ *M*_*m*_^*∗*^Δ.

**Theorem 4 .**

If *R*_0_ < 1, the disease-free periodic solution (*S*_*h*_^*∗*^(*t*), 0, *S*_*m*_^*∗*^(*t*), 0) is globally asymptotically stable. And if *R*_0_ > 1, it is unstable.

**Proof**

By Theorem 3 we obtain that if *R*_0_ < 1, (*S*_*h*_^*∗*^(*t*), 0, *S*_*m*_^*∗*^(*t*), 0) is locally stable. Next we prove that when *R*_0_ < 1 the disease-free solution (*S*_*h*_^*∗*^(*t*), 0, *S*_*m*_^*∗*^(*t*), 0) has global attractivity.

When *R*_0_ < 1 and by (iii) of Theorem 3, we have *ρ*(Φ_*F*−*V*_(*ω*)) < 1. So there exists a small enough constant *ε*_1_ > 0 such that *ρ*(Φ_*F*−*V*+*ε*_1_*N*_(*ω*)) < 1, where

(26) $$\begin{array}{c} N\left(\;t\;\right)=\left(\begin{array}{cc} 0 & \frac{\beta_{1}\left(t\right)}{1+\alpha_{1}\left(t\right)\left(S_{h}^{*}\left(t\right)+\varepsilon_{1}\right)}\\ \beta_{2}\left(t\right) & 0 \end{array}\right). \end{array}$$

From Lemma 2 and nonnegativity of the solutions, for any *ε*_1_ > 0 there exists *t*_1_ > 0 such that *S*_*h*_(*t*) ≤ *S*_*h*_^*∗*^(*t*) + *ε*_1_ and *S*_*m*_(*t*) ≤ *S*_*m*_^*∗*^(*t*) + *ε*_1_, so for all *t* > *t*_1_ we have

(27) Ih′t≤β1tSh∗t+ε1Imt1+α1tSh∗t+ε1−μ1tIht−υtIht,Im′t≤β2tSm∗t+ε1Iht−μ2tImt.

Considering the auxiliary system

(28) Ih′t~=β1tSh∗t+ε1Im′t~1+α1tSh∗t+ε1−μ1tIh′t~−υtIh′t~,Im′t~=β2tSm∗t+ε1Ih′t~−μ2tIm′t~.

From Lemma 1, it follows that there exists a positive *ω*-periodic solution *v*_1_(*t*) such that *J*(*t*) ≤ *e*^*pt*^*v*_1_(*t*), where *J*(*t*) = (*I*_*h*_(*t*), *I*_*m*_(*t*))^*T*^ and *p* = (1/*ω*)ln⁡*ρ*(Φ_*F*−*V*+*ε*_1_*N*_(*ω*)) < 0. Then lim_*t*→*∞*_*J*(*t*) = 0; that is, lim_*t*→*∞*_*I*_*h*_(*t*) = 0 and lim_*t*→*∞*_*I*_*m*_(*t*) = 0.

Moreover, from the equations of *S*_*h*_(*t*), *S*_*m*_(*t*), we get

(29) limt→∞Sht=Sh∗t,limt→∞Smt=Sm∗t.

Hence, disease-free periodic solution of system (1) is globally attractive. This completes the proof.

Define

(30) X=Sh,Ih,Sm,Im:Sh>0,  Ih≥0,  Sm≥0,  Im≥0,X0=Sh,Ih,Sm,Im∈X:Ih>0,  Im>0.

We have

(31) $$\begin{array}{c} \partial X_{0}=X\setminus X_{0}=\left\{\;\left(\;S_{h},I_{h},S_{m},I_{m}\;\right)\in X:I_{h}I_{m}=0\;\right\}. \end{array}$$

From system (1), it is easy to see that *X* and *X*_0_ are positively invariant, and ∂*X*_0_ is also a relatively closed set in *X*.

Let *P* : *X* → *X* be the Poincaré map associated with system (1), satisfying

(32) $$\begin{array}{c} P\left(\;x^{0}\;\right)=u\left(\;\omega ,x^{0}\;\right),\;\forall x^{0}\in X; \end{array}$$

*u*(*t*, *x*^0^) is the unique solution of system (1) satisfying initial condition *u*(0, *x*^0^) = *x*^0^. *P* is compact for the continuity of solutions of system (1) with respect to initial value, and *P* is point dissipative on *X*.

We further define

(33) M∂=Sh0,Ih0,Sm0,Im0∈∂X0:PmSh0,Ih0,Sm0,Im0∈∂X0  ∀m>0,

where *P*^*m*^ = *P*(*P*^*m*−1^) for all *m* > 1 and *P*^1^ = *P*. Now, prove

(34) $$\begin{array}{c} M_{\partial }=\left\{\;\left(\;S_{h}^{0},0,S_{m}^{0},0\;\right):S_{h}^{0}>0,\;\;S_{m}^{0}\geq 0\;\right\}. \end{array}$$

Obviously {(*S*_*h*_, 0, *S*_*m*_, 0) : *S*_*h*_ > 0, *S*_*m*_ ≥ 0}⊆*M*_∂_.

If *M*_∂_∖{(*S*_*h*_, 0, *S*_*m*_, 0) : *S*_*h*_ > 0, *S*_*m*_ ≥ 0} ≠ *∅*, then there exists at least a point (*S*_*h*_^0^, *I*_*h*_^0^, *S*_*m*_^0^, *I*_*m*_^0^) ∈ *M*_∂_ satisfying *I*_*h*_^0^ > 0 or *I*_*m*_^0^ > 0. We consider two possible cases.

If *I*_*h*_^0^ = 0 and *I*_*m*_^0^ > 0, then it is clear that from system (1) *I*_*m*_(*t*) ≥ 0 for any *t* > 0. From the second equation of system (1) and *S*_*h*_ > 0, we obtain

(35) IhtIh0e−∫0tμ1s+υsds+∫0tβ1sShsIms1+α1sShse∫stμ1τ+υτdτds>0,

for all *t* > 0.

If *I*_*m*_^0^ = 0 and *I*_*h*_^0^ > 0, then *I*_*h*_(*t*) = *I*_*h*_^0^*e*^−∫_0_^*t*^[*μ*_1_(*τ*)+*υ*(*τ*)]*dτ*^ > 0. From the third equation of system (1) and *S*_*m*_ > 0, we obtain

(36) ImtIm0e−∫0tμ2sds+∫0tβ2sSmsIhse∫stμ2τdτds>0,

for all *t* > 0. Hence, for any case, it follows that (*S*_*h*_(*t*), *I*_*h*_(*t*), *S*_*m*_(*t*), *I*_*m*_(*t*))∉∂*X*_0_, so (*S*_*h*_^0^, *I*_*h*_^0^, *S*_*m*_^0^, *I*_*m*_^0^) ∉ *M*_∂_. This leads to a contradiction; there exists one fixed point *E*_0_ = (*S*_*h*_^*∗*^(*t*), 0, *S*_*m*_^*∗*^(*t*), 0) of *P* in *M*_∂_.

In the following, we will discuss the uniform persistence of the disease, and *R*_0_ serves as a threshold parameter for the extinction and the uniform persistence of the disease.

**Theorem 5 .**

If *R*_0_ > 1, then system (1) is uniformly persistent. There exists a positive constant *ε*, such that for all initial conditions (1) satisfies

(37) limt→∞inf⁡Iht≥ε,limt→∞inf⁡Imt≥ε.

When *R*_0_ > 1, system (1) admits at least one positive periodic solution.

**Proof**

From Theorem 3, if *R*_0_ > 1 then we obtain *ρ*(Φ_*F*−*V*_(*ω*)) > 1. For an arbitrary small constant *η* > 0, that *ρ*(Φ_*F*−*V*−*ηN*_(*ω*)) > 1, *N*(*t*) is the same as in Theorem 3. From assumption (*H*_*2*_), we obtain any small enough *ε* > 0, ∫_0_^*ω*^[*r*(*t*) − *α*(*t*)*ε*]*dt* > 0. Consider perturbed equations

(38) Sεh′t=Λt−εβ1tSεht1+α1tSεht−μ1tSεht,

(39) Sεm′t=rtSεmt1−SεmtKt−εβ2tSεmt.

Using Lemma 2 in [25] and Lemma 1 of [27], we obtain (38) and (39) that admit globally uniformly attractive positive *ω*-periodic solutions *S*_*εh*_^*∗*^(*t*) and *S*_*εm*_^*∗*^(*t*). For the continuity of solutions with respect to *ε*, and for *η* > 0 there exists *ε*_1_ > 0 for all *t* ∈ [0, *ω*]; thus we have

(40) Sε1m∗t>Sm∗t−η,Sε1h∗t>Sh∗t−η.

Denote *x*^0^ = (*S*_*h*_^0^, *I*_*h*_^0^, *S*_*m*_^0^, *I*_*m*_^0^) ∈ *X*_0_, according to the continuity of the solution with respect to the initial condition; there exists *δ* for given *ε*_1_, for all *x*^0^ ∈ *X*_0_ with ‖*x*^0^ − *E*_0_‖ < *δ*; it follows ‖*u*(*t*, *x*^0^) − *u*(*t*, *E*_0_)‖ < *ε*_1_ for all *t* ∈ [0, *ω*].

Following, we prove

(41) $$\begin{array}{c} \underset{m\to \infty }{lim}\sup d\left(\;P^{m}\left(\;x^{0}\;\right),E_{0}\;\right)\geq \delta . \end{array}$$

We suppose the conclusion is not true; then following inequality holds:

(42) $$\begin{array}{c} \underset{m\to \infty }{lim}\sup d\left(\;P^{m}\left(\;x^{0}\;\right),E_{0}\;\right)<\delta , \end{array}$$

for some *x*^0^ ∈ *X*_0_. Without loss of generality, we can assume that

(43) $$\begin{array}{c} d\left(\;P^{m}\left(\;x^{0}\;\right),E_{0}\;\right)<\delta \;\forall m\geq 0. \end{array}$$

So we obtain

(44) ut,Pmx0−ut,E0<ε1∀m≥0,  t∈0,ω.

For any *t* ≥ 0,  *t* = *mω* + *t*′, where *t*′ ∈ [0, *ω*] and *m* = [*t*/*ω*] is the greatest integer less than or equal to *t*/*ω*, so we have

(45) ut,x0−ut,E0ut′,Pmx0−ut′,E0<ε,∀t≥0.

Hence, it follows that 0 ≤ *I*_*h*_(*t*) ≤ *ε*_1_ and 0 ≤ *I*_*m*_(*t*) ≤ *ε*_1_ for all *t* ≥ 0. Then from the first and third equations of (1),

(46) Sh′t≥Λt−ε1β1tSht1+α1tSht−μ1tSht,Sm′t=rtSmt1−SmtKt−ε1β2tSmt.

By the comparison principle, we obtain for any *t* ≥ 0

(47) Sht≥Sε1ht,Smt≥Sε1mt.

Consider (38); there exists *t*_1_ > 0; for all *t* > *t*_1_ we have

(48) Sε1mt>Sε1m∗t−η,Sε1ht>Sε1h∗t−η.

By (38) and (48) we obtain

(49) Smt>Sm∗t−η,Sht>Sh∗t−η.

Then for all *t* > *t*_1_ we have

(50) Iht≥β1tSh∗t−ηImt1+α1tSh∗t−η−μ1tIht−υtIht,Imt≥β2tSm∗t−ηIht−μ2tImt.

Consider the following auxiliary system:

(51) Iht¯=β1tSh∗t−ηImt¯1+α1tSh∗t−η−μ1tIht¯−υtIht¯,Imt¯=β2tSm∗t−ηIht¯−μ2tImt¯.

From Lemma 1, it follows that there exists a positive *ω*-periodic function *v*_2_(*t*) such that (51) has a solution *J*(*t*) = *v*_2_(*t*)*e*^*p*_1_*t*^, where *p*_1_ = (1/*ω*)ln⁡(*ρ*(Φ_*F*−*V*−*ηN*_(*ω*))). For *ρ*(Φ_*F*−*V*−*ηN*_(*ω*)) > 1,

(52) limt→∞Iht=+∞,limt→∞Imt=+∞.

This leads to a contradiction.

That is to say, *M*_∂_∖{(*S*_*h*_, 0, *S*_*m*_, 0) : *S*_*h*_ > 0, *S*_*m*_ ≥ 0} = *∅* and {*M*_1_} is globally attractive in *M*_∂_, and all orbit in *M*_∂_ converges to {*M*_1_}. By [22], we obtain that *P* is weakly uniformly persistent with respect to (*X*_0_, ∂*X*_0_). All solutions are uniformly persistent with respect to (*X*_0_, ∂*X*_0_); thus we have lim_*t*→*∞*_*I*_*h*_(*t*) ≥ *ε*,  lim_*t*→*∞*_*I*_*m*_(*t*) ≥ *ε*.

## 4. Sensitivity Analysis and Prevention Strategy

We conducted numerical simulation to this model and computed the reproductive numbers *R*_0_. It was confirmed that using the basic reproduction number of the time-averaged autonomous systems of a periodic epidemic model overestimates or underestimates infection risks in many other cases. Bacaer and Guernaoui give methods to compute *R*_0_, such as method of discretization of the integral eigenvalue [36] and Fourier series method for general periodic case and sinusoidal case and application of Floquet Theory method [37]. In [22] Wang and Zhao propose that in order to compute *R*_0_ we only need to compute the spectrum of evolution operator of the following system (53):

(53) $$\begin{array}{c} \frac{dw}{dt}=\left[\;-V\left(\;t\;\right)+\frac{F\left(t\right)}{\lambda }\;\right]w,\;w\in R^{n},\;\;\lambda \in \left(0,\infty \right); \end{array}$$

here system (53) is *ω*-periodic equation, and *W*(*t*, *s*, *λ*) is the evolution operator of system (53) with *t* ≥ *s*, *s* ∈ *R*. By Perron-Frobenius theorem *ρ*(*W*(*ω*, 0, *λ*)) is an eigenvalue of *W*(*t*, 0, *λ*), *t* ≥ 0. Next, using Theorem 2.1 in [22] to compute *R*_0_ numerically, *R*_0_ serves as threshold parameter in periodic circumstances.

**Figure 1:**
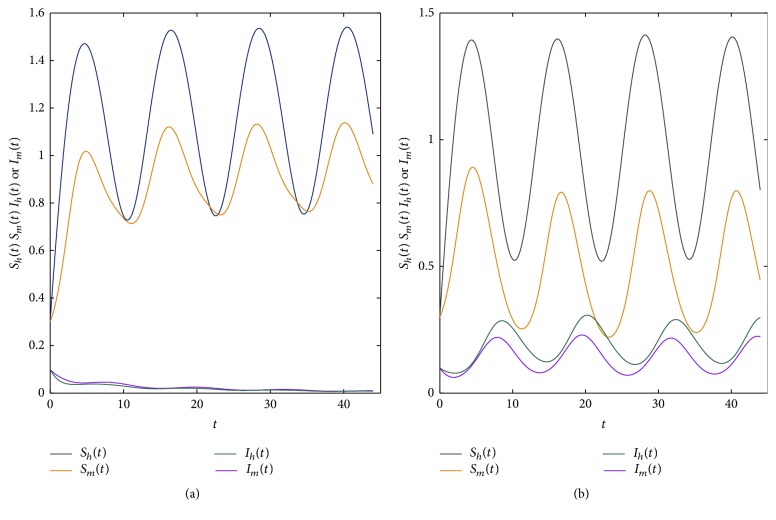
Plot the evolution tendency of four populations. (a) Fixed parameters *β*_1_(*t*) = 0.6 + 0.1sin⁡(2*πt*/12),  *β*_2_(*t*) = 0.7 + 0.1sin⁡(2*πt*/12); then *R*_0_ = 0.9243 < 1; (b) Parameters *β*_1_(*t*) = 0.9 + 0.1sin⁡(2*πt*/12),  *β*_2_(*t*) = 1.2 + 0.1sin⁡(2*πt*/12); then *R*_0_ = 1.4662 > 1.

**Figure 2:**
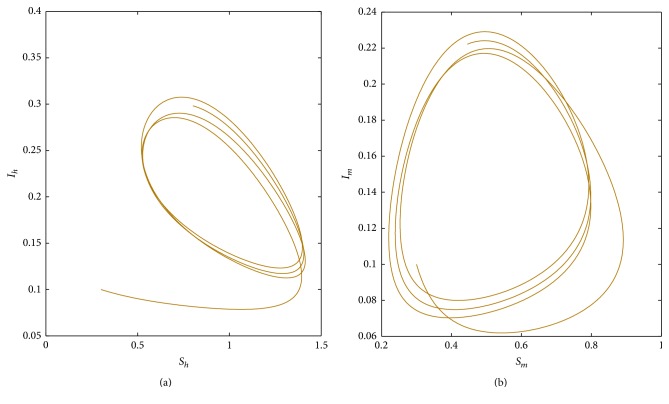
When *R*_0_ = 1.4662, we graph the trajectory of two populations in spaces (*S*_*h*_, *I*_*h*_) and (*S*_*m*_, *I*_*m*_), respectively.

Firstly, by the means of the software Matlab we compute *R*_0_. We choose parameters Λ(*t*) = 0.6 + 0.4sin⁡(2*πt*/12), *μ*_1_(*t*) = 0.5 + 0.1sin⁡(2*πt*/12), *μ*_2_(*t*) = 0.8 + 0.1sin⁡(2*πt*/12), *β*_1_(*t*) = 0.6 + 0.1sin⁡(2*πt*/12), *β*_2_(*t*) = 0.7 + 0.1sin⁡(2*πt*/12), *α*_1_(*t*) = 0.2 + 0.1sin⁡(2*πt*/12), *υ*(*t*) = 0.02 + 0.03sin⁡(2*πt*/12), *r*(*t*) = 0.5 + 0.4sin⁡(2*πt*/12), *K*(*t*) = 0.9 + 0.3sin⁡(2*πt*/12). By numerical calculations, we obtain *R*_0_ = 0.9243 < 1; then the disease will be extinct; see Figure 1(a). If we choose *β*_1_(*t*) = 0.9 + 0.1sin⁡(2*πt*/12), *β*_2_(*t*) = 1.2 + 0.1sin⁡(2*πt*/12), then *R*_0_ = 1.4662 > 1; the disease is permanent; see Figure 1(b). The evolution trajectory in spaces (*S*_*h*_, *I*_*h*_) and (*S*_*m*_, *I*_*m*_) are in Figures 2(a) and 2(b), respectively.

In order to perform sensitivity analysis of parameters *β*_1_(*t*), *β*_2_(*t*), *K*(*t*), and *α*_1_(*t*), we fix all parameters as in Figure 1, except that we choose the composite functions as follows:

(54) β1t=β01+0.1sin⁡2πt12,β2t=β02+0.1sin⁡2πt12,Kt=k0+0.3sin⁡2πt12,α1t=α0+0.1sin⁡2πt12,

where *β*_01_ = (1/12)∫_0_^12^*β*_1_(*t*)*dt*,  *β*_02_ = (1/12)∫_0_^12^*β*_2_(*t*)*dt*,  *k*_0_ = (1/12)∫_0_^12^*K*(*t*)*dt*, and *α*_0_ = (1/12)∫_0_^12^*α*_1_(*t*)*dt*.

**Figure 3:**
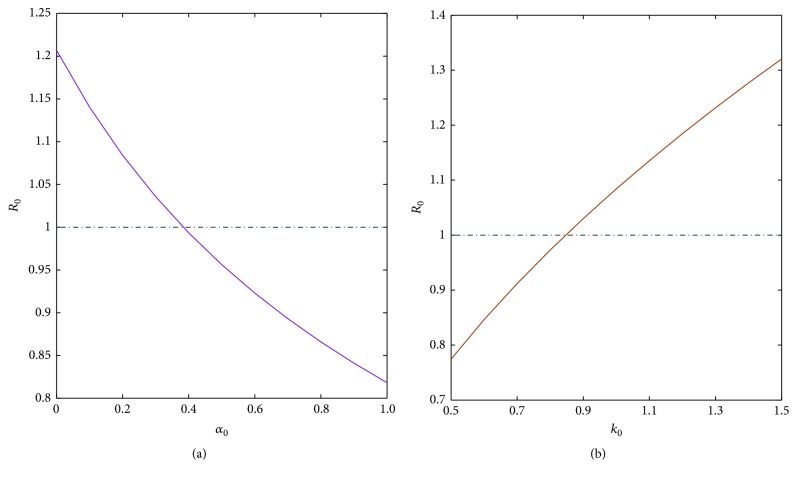
Sensitivity analysis of the basic reproduction *R*_0_ with parameter *k*_0_ or *α*_0_.

We first fix other parameters and detect the effect of parameters of *k*_0_ and *α*_0_ on *R*_0_. From Figure 3(a), we see that with the increase of *α*_0_, *R*_0_ decreases, and the gradient also decreases, so this strengthens the psychological hint of susceptible human individuals to be benefit for the extinction of the disease. In Figure 3(b), with the increasing of *k*_0_ the sensitivity of *R*_0_ increases. That is to say, the carrying capacity of environment for mosquito is bigger and the disease is widespread more easily, so decreasing the circumstance fit survival for mosquitoes, such as contaminated pool or puddle and household garbage, is a necessary method for the extinction of disease.

**Figure 4:**
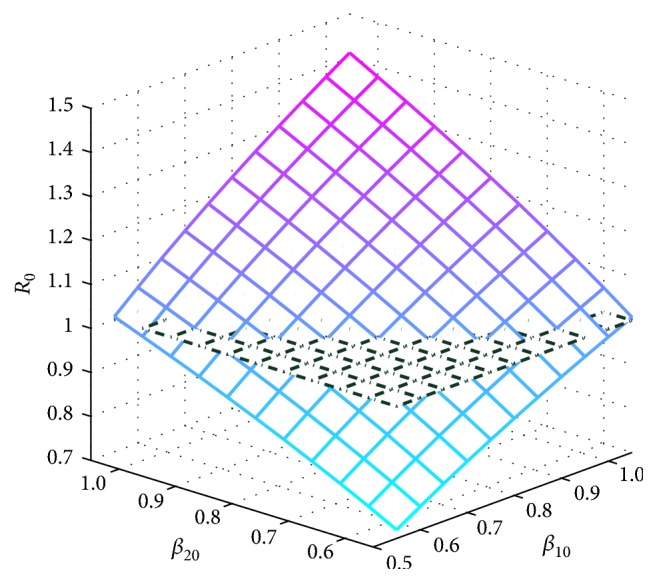
Sensitivity analysis of the basic reproduction *R*_0_ with parameters *β*_01_ and *β*_02_.

Next, we consider the combined influence of parameters *β*_10_ and *β*_20_ on *R*_0_; in Figure 4 we can see that the basic reproduction number *R*_0_ may be less than 1 when *β*_10_ and *β*_20_ are small; the smaller *β*_20_ the more sensitive the effect on *R*_0_.

**Figure 5:**
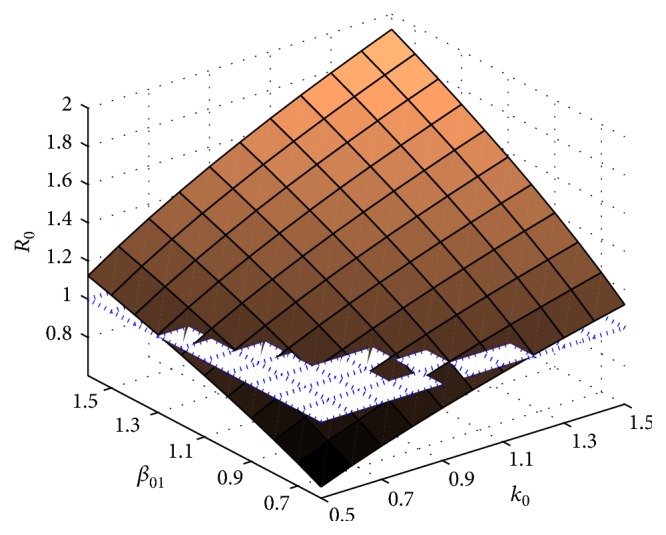
Sensitivity analysis of the basic reproduction *R*_0_ with parameters *β*_01_ and *k*_0_.

In Figure 5, the basic reproduction number *R*_0_ is affected by *β*_10_ and *k*_0_; with the increasing of *k*_0_ the sensitivity of *R*_0_ increases; if we fix *β*_10_ as a constant the case will be similar to Figure 3(b). And the similar trend of *β*_10_ on the sensitivity of *R*_0_, so in the season in which temperature and humidity are more beneficial for mosquito population to give birth and propagate taking measures to avoid more bites is necessary.

## 5. Conclusion

In this paper, we have studied the transmission of lymphatic filariasis; lymphatic filariasis is a mosquito-borne parasitic infection that occurs in many parts of the developing world. In order to systematically investigate the impact that vector genus-specific dependent processes may have on overall lymphatic filariasis transmission, we, according to the nature characteristic of lymphatic filariasis and considering the logistic growth in periodic environments of mosquito, model the transmission of lymphatic filariasis. The dynamic behavior of system (1) is determined by the threshold parameter *R*_0_; when *R*_0_ < 1 disease-free periodic solution is globally asymptotically stable and when *R*_0_ > 1 disease is uniformly persistent. We also give some numerical simulations which support the results we prove, confirming that *R*_0_ serves as a threshold parameter. Sensitivity analysis show effects of parameters on *R*_0_, which contribute to providing a decision support framework for determining the optimal coverage for the successful prevention programme.
